# Editorial: Optimizing fertilizer and irrigation for specialty crops using precision agriculture technologies

**DOI:** 10.3389/fpls.2026.1760142

**Published:** 2026-01-20

**Authors:** Amir Ali Khoddamzadeh, Sukhbir Singh, Maruthi Sridhar Balaji Bhaskar

**Affiliations:** 1 Department of Earth and Environment, Institute of Environment, Florida International University, Miami, FL, United States; 2 Department of Plant and Soil Science, College of Agricultural Sciences and Natural Resources, Texas Tech University, Lubbock, TX, United States

**Keywords:** irrigation management, optical sensors, precision agriculture, remote sensing, specialty crops, fertilizer management

## Introduction

Global demand for specialty crops continues to grow, driven by consumer preferences for high-quality, diverse produce and by the need to transition toward environmentally sustainable production systems (Vuppalapati, 2023). At the same time, specialty crop agriculture faces mounting constraints: increasing pressure on water and nutrient resources, spatial heterogeneity of soils and microclimates, stricter environmental regulations, and the imperative to improve both yield and quality under variable climatic conditions (Sandhu et al., 2025; Gonzalez et al., 2025). Precision agriculture offers a pathway to address these challenges through site-specific, data-driven management of fertilizer and irrigation. By integrating proximal and remote sensors, advanced analytics, and decision-support tools, precision approaches can enhance resource-use efficiency, reduce nutrient losses, and improve crop performance while safeguarding environmental quality (Munoz Salas et al., 2025; Souza Costa and Khoddamzadeh, 2025).

This Research Topic brings together studies that collectively address three interrelated challenges: (i) how to diagnose crop nutrient and water status at high spatial and temporal resolution; (ii) how to translate these diagnostics into variable-rate management of fertilizers and irrigation; and (iii) how to integrate technological, biological, and modeling approaches into coherent decision-support systems for specialty crops.

## Sensor-based and remote sensing approaches for nutrient and water diagnostics

Several contributions demonstrate how proximal and remote sensing technologies enable non-destructive, spatially explicit assessment of crop nutritional and water status. Costa and Khoddamzadeh employ optical sensors (GreenSeeker™, SPAD, and atLEAF) to determine nitrogen requirements for Satinleaf (Chrysophyllum oliviforme), illustrating how sensor indices can guide site-specific nitrogen application in horticultural systems. Kong et al. extend this concept using UAV-based hyperspectral imaging combined with machine-learning models to estimate leaf chlorophyll content in banana, integrating spectral and textural features to map nutrient status across heterogeneous orchards.

Water status monitoring is similarly advanced by Ding et al., who apply UAV hyperspectral imagery and machine learning to estimate potato canopy leaf water content across growth stages, enabling stage-specific irrigation decisions. Huang et al. combine UAV multispectral data with ground-based SPAD measurements to generate spatially explicit nitrogen diagnostic maps for orchard systems. Collectively, these studies show that sensor-driven diagnostics can replace uniform input strategies with real-time, crop-responsive management. Their convergence on data fusion (spectral and structural features) and predictive modeling highlights a broader shift toward operational remote sensing as a core component of precision nutrient and water management.

## Soil and geophysical mapping for site-specific management

Understanding within-field variability in soil properties is essential for variable-rate input management. Scudiero et al. integrate apparent soil electrical conductivity with gamma-ray spectrometry to characterize particle-size distribution in micro-irrigated citrus orchards. Their approach enables delineation of management zones based on water-holding capacity and nutrient retention, supporting targeted irrigation and fertilization. This work emphasizes that crop-based sensing must be complemented by soil and geophysical characterization to establish the physical context in which nutrient and water decisions are made. Integrating soil mapping with canopy level diagnostics is key to robust site-specific management.

## Organic and bio-based fertilizers in precision nutrient management

A set of studies explores how organic amendments and biofertilizers can be integrated into precision frameworks to reduce reliance on synthetic inputs. Yang et al. report that substituting silkworm excrement in compound fertilizers increases bamboo shoot yield and enhances soil microbial communities in Phyllostachys edulis forests. Mingjing et al. demonstrate that castor bean meal based biofertilizers improve growth, yield, and quality of Tartary buckwheat. Huang et al. show that partial replacement of synthetic nitrogen with organic sources enhances aroma-related metabolites in Wuyi Rock tea, linking nutrient management to product quality. Zhao et al. further document improvements in soil physicochemical properties and cotton yield following organic fertilizer inputs in southern Xinjiang. These studies collectively indicate that organic and bio-based fertilizers can be deployed in precision systems to achieve dual goals: improving soil health while maintaining or enhancing crop performance. Their integration with sensor-based diagnostics offers a promising route toward environmentally sustainable, input efficient specialty crop production.

## Optimizing irrigation–fertilization regimes and cropping systems

Several contributions address coordinated management of water and nutrients. Zhang et al. identify optimal fertilizer rates and sowing densities that maximize yield, quality, and nutrient-use efficiency in oats. Gao et al. demonstrate that synchronized irrigation and fertilization in maize mung bean intercropping enhances photosynthetic efficiency, water use, and yield. Hutchinson et al. compare sensor-controlled fertigation with timer-based systems in hydroponic strawberry production, showing that real-time moisture sensing improves resource and energy efficiency. Guo et al. provide a meta-analysis for kiwifruit, quantifying how irrigation and fertilization strategies affect yield, water-use efficiency, and fruit quality across environments. These studies underscore the importance of coupling irrigation and fertilization decisions rather than optimizing them in isolation. Precision management emerges not only as a technological upgrade but as a systems approach to coordinating multiple inputs for maximum agronomic and environmental benefit.

## Modeling, meta-analysis, and decision-support tools

Beyond field-level experimentation, modeling and synthesis approaches contribute to scalable decision-making. Tan et al. implement the APSIM crop model to simulate winter wheat growth dynamics, demonstrating the value of “digital twin” frameworks for predicting biomass, phenology, and yield under variable climate and management scenarios. Yang et al. apply meta-analysis to compare ratoon-season and main crop cereals, revealing improvements in grain quality under optimized water nutrient regimes. Modeling and meta-analytic approaches provide the temporal and spatial generalization needed to translate site-specific findings into broadly applicable management guidelines. When coupled with sensor-derived data streams, these tools form the backbone of adaptive, data driven agronomic decision systems.

## Toward integrated precision nutrient water management

Taken together, the contributions in this Research Topic illustrate a transition from isolated technological applications to integrated management frameworks. Sensor networks diagnose crop and soil status; organic and bio-based fertilizers enhance sustainability; coordinated irrigation fertilization regimes optimize resource use; and models synthesize data across scales. The emerging paradigm is one of adaptive precision agriculture, in which real-time diagnostics, biological inputs, and predictive analytics are combined to deliver site-specific, environmentally responsible management. Future progress will depend on: (i) tighter integration of multi-sensor platforms with crop and soil models; (ii) standardization and interoperability of agronomic data; (iii) incorporation of artificial intelligence for real-time optimization; and (iv) development of scalable solutions accessible to both high-tech operations and resource-limited producers.

## Conclusions

This Research Topic demonstrates that precision agriculture technologies can substantially improve fertilizer and irrigation management in specialty crops by enhancing resource-use efficiency, crop quality, and environmental performance. By uniting sensor-based diagnostics, soil mapping, organic nutrient strategies, coordinated water-nutrient management, and modeling tools, the collected studies move beyond incremental optimization toward integrated, system-level solutions. Looking ahead, continued innovation in sensor technologies, data analytics, and decision-support systems coupled with close collaboration among researchers, growers, and technology providers will be essential to realize the full potential of precision agriculture. Such integration will ensure that specialty crop production remains productive, profitable, and sustainable under increasingly complex environmental and market conditions.

## References

[B1] Gonzalez P. Tucker D. Maruthi B. B. Sridhar Nageswara-Rao R. Balaji Baskar M. S. Griffith P. . (2025). Enhancing cabbage palm resilience to saltwater stress through silicon applications. HortScience 60, 1547–1554. doi: 10.21273/HORTSCI18718-25

[B2] Munoz Salas M. Roddy A. B. PDastpak A. Costa B. N. S. Khoddamzadeh A. A. (2025). Ecophysiological adaptations of musa haekkinenii to light intensity and water quality. Special issue: management of artificial light in horticultural crops. Horticulture 11, 1188. doi: 10.3390/horticulturae11101188

[B3] Sandhu D. Pudussery E. Tammar K. Cendan L. Khoddamzadeh A. A. Ferreira J. (2025). Decoding the genetic basis of salinity tolerance in tomatoes through ion transport and stress regulation. ACS Agric. Sci. Technol. 5, 1817–1826. doi: 10.1021/acsagscitech.5c00209

[B4] Souza Costa B. Khoddamzadeh A. A. (2025). Data-driven nitrogen application for Satinleaf: Leveraging optical sensors in urban landscape management. Technical Advances in Plant Science. Special Issue: Optimizing Fertilizer and Irrigation for Specialty Crops Using Precision Agriculture Technologies. Front. Plant Sci. 16. doi: 10.3389/fpls.2025.1522662, PMID: 39980485 PMC11839651

[B5] Vuppalapati C. (2023). “ Specialty crops,” in Specialty crops for climate change adaptation: Strategies for enhanced food security by using machine learning and artificial intelligence ( Springer Nature Switzerland, Cham), 35–197.

